# Editorial: Dynamic roles of anxiety and motivation in second/foreign language acquisition

**DOI:** 10.3389/fpsyg.2023.1145368

**Published:** 2023-02-15

**Authors:** Meihua Liu, Chin-Hsi Lin, Yining Zhang

**Affiliations:** ^1^Department of Foreign Languages and Literatures, Tsinghua University, Beijing, China; ^2^Faculty of Education, The University of Hong Kong, Pokfulam, Hong Kong SAR, China

**Keywords:** anxiety, motivation, dynamic, second/foreign language learning, interaction

Motivation and anxiety, which are classified both as psychological and as affective factors, have been shown to play important roles in second-/foreign-language (SL/FL) learning at all levels. Specifically, students with higher motivation tend to study their target languages (TLs) more effectively, and those with high anxiety, less effectively. Additionally, scholars agree that SL/FL motivation and anxiety (1) are closely related to each other; (2) interact with other variables to affect SL/FL learning; and (3) are dynamic, and thus that their effects on SL/FL learning are also dynamic. Nevertheless, in light of the complex nature of language learning and the huge diversity of SL/FL learning populations and contexts, both motivation and anxiety remain under-researched. Conducted in diverse contexts, the 15 articles making up this special issue expand research on anxiety, motivation, and their relations with SL/FL learning both theoretically and methodologically.

Of these 15 articles, two are reviews (Gao; Wang and Xue) of research on anxiety and motivation, respectively. Wang and Xue focus on how the expectancy-value motivational model impacts academic motivation, engagement, participation in educational tasks, and academic performance. Gao classifies existing FL-anxiety scales into three types: test-based, measuring speaking anxiety; classroom-based, measuring speaking anxiety; and activity-based, also measuring speaking anxiety. She also introduces Classical Testing Theory and Rasch measurement as two major statistical paradigms for guaranteeing the reliability of these scales. As well as summarizing the emerging themes of the relevant research, the author discusses the dynamic approach to interpreting the interrelationship of anxiety, language performance, and other factors involved in language learning, and highlights possible directions for future anxiety-related research.

The remaining 13 papers all report on empirical research: five using mixed methods (He et al.; Lin; Rasool et al.; Ren and Abhakorn; Yan and Liang); four, quantitative methods (Dong, Liu et al.; Greenwald et al.; Wu et al.; Zhang and Dong); one, qualitative methods (Lu and Yoon); and the remaining three, experimental designs (Dong, Liu et al.; Izadpanah; Rezaee and Seyri). They examine various aspects of anxiety and motivation including theory development, strategies, measurements, effects, and sources.

Three of the papers focus on anxiety. Yan and Liang's contribution investigates the effects of English-Chinese interpretation-classroom foreign language anxiety (ICFLA) on interpretation learning and dependency distance (DD) among 49 undergraduate and graduate students in Hong Kong. They report a significant negative correlation between ICFLA levels and consecutive interpretation achievement scores. ICFLA was also negatively correlated with DD in consecutive interpretations. Rasool et al.'s article explores levels of and reasons for writing anxiety, and gender influence on anxiety levels, among 72 pre-service FL teachers in Pakistan. It reports that most of the participants experienced medium to high writing-anxiety levels, without gender differences, that were accounted for by linguistic challenges, fear of negative judgment, lack of self-confidence, and bad prior experiences. Lin's contribution uses interview and survey data from 243 Chinese students of L3 French at a university in the UK to explore the relationship between their L3 anxiety and their self-efficacy, which emerged as negative. It also shows that grammatical and pronunciation similarities between English—the participants' L2—and French positively decreased these students' anxiety levels.

Three further contributions focus on motivation. Ren and Abhakorn's explores the psychological and cognitive factors behind college students' loss of motivation to learn English in universities in China, and the interrelationships of those factors. Specifically, they constructed a shopping-cart model based on the results of 23 interviews and used structural equation modeling to test it on questionnaire data from 286 demotivated students. This revealed three distinct pathways whereby the respondents were demotivated: (1) a large discrepancy between their actual and required positioning of English learning, (2) a low required positioning of English learning, and (3) a low value of English learning in students' minds. He et al. also studied Chinese university students, collecting interview and survey data from 79 of them and investigating their perceptions and practices of rubric use throughout a task process. Their results highlight the important roles of trait motivation and task motivation in the effectiveness of rubric use during assessment. Greenwald et al.'s contribution, meanwhile, looks at intrinsic and extrinsic motivation among 851 monolingual and 196 bilingual children in the United States, and suggests that among the latter group, these two types of motivation are not antagonistic—unlike with their monolingual counterparts.

Four studies examined the interaction of anxiety and motivation. Dong, Liu et al.'s based on a questionnaire survey of 280 Chinese high school students, explores the relations among FL classroom anxiety (FLCA), enjoyment (FLE), and expectancy-value motivation, as well as how effectively these three variables predict students' self-rated FL proficiency. It reports that (1) the students' FLE was significantly and positively correlated with all dimensions of expectancy-value motivation, whereas their FLCA and expectancy-value motivation demonstrated a complex correlation pattern; and (2) expectancy beliefs, intrinsic value, private enjoyment of FL learning and anxiety arising from fear of negative evaluation jointly and significantly predicted the students' self-rated FL proficiency. Dong, Jamal Mohammed et al. report on their exploration, *via* a pre- and post-test experimental design, of the effects of three instructional modes—computer-assisted language learning (CALL), mobile-assisted language learning (MALL), and face-to-face (FTF) learning—on Iranian EFL learners' motivation, anxiety and self-efficacy. Specifically, they randomly assigned 30 such learners to each of three classes, each comprising 25 1-h sessions, and found that the experimental groups' motivation, anxiety, and self-efficacy were positively affected by CALL-based and MALL-based instruction, though there was no statistically significant difference between the CALL and MALL groups in this regard. Zhang and Dong's paper examines how 230 Chinese college students' motivational-regulation strategies affected their proximal and distal L2 writing-achievement emotions (i.e., enjoyment and anxiety), and tested for possible interactive effects of such strategies and self-regulated learning strategies on the same two emotions. They report that all the motivational-regulation strategies that they studied directly predicted both proximal and distal writing enjoyment, but that only a performance-oriented one predicted proximal or distal writing anxiety. Another key finding is that a social-behavior learning strategy counteracted the high proximal anxiety caused by heavy use of the performance self-talk motivational-regulation strategy. Moreover, it highlights motivational-regulation strategies as stable predictors of both proximal and distal writing wellbeing. Wu et al.'s contribution, based on a sample of 223 students of the Top-Notch Students of Basic Disciplines Training Program in a top Chinese university, examines English-use anxiety (EUA), motivation, self-efficacy, and use of English, along with these variables' predictive effects. Their findings indicate that (1) in general, EUA and language-learning orientation were significantly and negatively correlated, and significantly but positively correlated with the other measured variables; (2) the participants' EUA and intrinsic-motivation knowledge significantly predicted their English achievement; and (3) their use of English and self-efficacy mediated the effects of EUA and language-learning orientations on their English achievement.

The remaining three studies focused on other issues related to motivation. Lu and Yoon's paper reports on how they used interview, textual and documentary data to examine the influence of power relations on the research practices of six EFL academics at a Chinese university, as well as the same individuals' coping strategies. They conclude that, even though their participants were driven to engage in research by a combination of intrinsic and extrinsic motivations, their research endeavors were undermined by the marginalized status of EFL researchers from non-elite universities, as imposed by the Chinese academic circle. Even so, they exerted their agency *via* micropolitical literacy and tried to seek ways out of their unfavorable academic culture. Izadpanah's contribution involves a pre- and post-test experimental study of 354 high-school students, aimed at ascertaining the impact of flipped teaching (FT) on EFL students' academic resilience (AR), self-directed learning (SDL), and learner autonomy (LA). It shows that FT significantly affected AR, SDL, and LA, and that the mean scores of EFL students' AR, SDL, and LA were higher through FT. Last but not least, Rezaee and Seyri's piece examines boredom as experienced by 84 online students of English for academic purposes and the success of an autonomy-oriented intervention program aimed at alleviating such boredom.

Collectively and individually, these studies shed considerable light on teaching and learning, both theoretically and empirically. Nevertheless, more research on anxiety and motivation is still warranted. For example, more longitudinal studies are needed to document changes in people's anxiety and motivation, and such changes' effects on SL/FL learning. Likewise, there needs to be more research on both technological and non-technological strategies for intervening to reduce anxiety and increase motivation in the context of such; and anxiety and motivation connected with the learning of SLs/FLs other than English remain under-researched.

## Author contributions

ML drafted the editorial. C-HL and YZ revised and polished the editorial. All authors contributed to the article and approved the submitted version.

